# Continuous subcortical language mapping in awake glioma surgery

**DOI:** 10.3389/fonc.2022.947119

**Published:** 2022-08-12

**Authors:** Hans W. Axelson, Francesco Latini, Malin Jemstedt, Mats Ryttlefors, Maria Zetterling

**Affiliations:** ^1^ Department of Medical Sciences, Section of Clinical Neurophysiology, Uppsala University, Uppsala, Sweden; ^2^ Department of Medical Sciences, Section of Neurosurgery, Uppsala University, Uppsala, Sweden; ^3^ Department of Medical Sciences, Speech-Language Pathology, Uppsala University, Uppsala, Sweden

**Keywords:** awake craniotomy, subcortical electrical stimulation, short-train stimulation, monopolar stimulation, intraoperative language testing, tractography

## Abstract

Repetitive monopolar short-train stimulation (STS) delivered from a suction probe enables continuous mapping and distance assessment of corticospinal tracts during asleep glioma resection. In this study, we explored this stimulation technique in awake glioma surgery. Fourteen patients with glioma involving language-related tracts were prospectively included. Continuous (3-Hz) cathodal monopolar STS (five pulses, 250 Hz) was delivered *via* the tip of a suction probe throughout tumor resection while testing language performance. At 70 subcortical locations, surgery was paused to deliver STS in a steady suction probe position. Monopolar STS influence on language performance at different subcortical locations was separated into three groups. Group 1 represented locations where STS did not produce language disturbance. Groups 2 and 3 represented subcortical locations where STS produced language interference at different threshold intensities (≥7.5 and ≤5 mA, respectively). For validation, bipolar Penfield stimulation (PS; 60 Hz for 3 s) was used as a “gold standard” comparison method to detect close proximity to language-related tracts and classified as positive or negative regarding language interference. There was no language interference from STS in 28 locations (Group 1), and PS was negative for all sites. In Group 2 (STS threshold ≥ 7.5 mA; median, 10 mA), there was language interference at 18 locations, and PS (median, 4 mA) was positive in only one location. In Group 3 (STS threshold ≤ 5 mA; median, 5 mA), there was language interference at 24 locations, and positive PS (median 4 mA) was significantly (p < 0.01) more common (15 out of 24 locations) compared with Groups 1 and 2. Despite the continuous stimulation throughout tumor resection, there were no seizures in any of the patients. In five patients, temporary current spread to the facial nerve was observed. We conclude that continuous subcortical STS is feasibly also in awake glioma surgery and that no language interference from STS or interference at ≥7.5 mA seems to indicate safe distance to language tracts as judged by PS comparisons. STS language interference at STS ≤ 5 mA was not consistently confirmed by PS, which needs to be addressed.

## Introduction

The extent of resection of low-grade gliomas is positively correlated with survival (1–4). To preserve neurological functions, awake surgery in combination with electrostimulation (mapping) is recommended (5, 6). The bipolar Penfield stimulation (PS) technique (7) is currently the recommended stimulation technique for localizing cortical and subcortical language-related areas in awake glioma surgery (8, 9). PS was previously also the first choice for asleep mapping of the primary motor cortex and its corticospinal tracts (10). For this purpose, however, the monopolar short-train stimulation (STS) technique (11, 12) seems to be a better alternative (12, 13). Of particular relevance for this study is that subcortical monopolar STS enables “proximity alerts” with a millimeter resolution as the stimulation strength (mA) required to obtain motor evoked potentials (MEPs) correlates to the distance to the corticospinal tracts (14, 15). Further, it is possible to continuously assess the distance to subcortical motor tracts during tumor resection by delivering repetitive STS *via* a suction probe (16). As for *language* mapping, a few previous studies (17–19) provide support that monopolar STS is useful also for language mapping in awake craniotomy. Originally, the STS technique was introduced in awake surgery to offer a less epileptogenic stimulation technique than PS (17, 20). It is noteworthy that continuous subcortical “dynamic mapping” (16), i.e., the technique used in this study, was recently suggested for language mapping (21), although there seems to be a lack of published experience with this stimulation method in awake surgery.

The main aim of this study was to explore whether the minimum STS current intensity required to produce language interference provides information about the distance to language-related tracts. For validation purposes, we compared results from monopolar STS with the well-established bipolar PS subcortical mapping technique and information from diffusion tensor imaging (DTI). The purpose was not to compare different stimulation protocols in detail (i.e., monopolar STS vs. bipolar PS) but to explore whether *continuous* subcortical STS from a suction probe is useful also for subcortical language mapping (which is already established for subcortical motor mapping). If so, this may allow for uninterrupted awake surgery as long as the stimulation does not produce errors in concurrent language testing at lower stimulation intensities. In parallel, we documented possible side effects (e.g., seizures) considering that the patient is exposed to a long period (> 1 h) of almost persistent electrical stimulation.

## Methods

### Patient selection and information

Fourteen patients with a radiological suspicion of a low-grade glioma in close connection to eloquent language areas were prospectively included from August 2018 to February 2020. The study was approved by the institutional ethics review board (2015/210/2). Informed consent was obtained prior to surgery at the Department of Neurosurgery, Uppsala University Hospital. This patient material has previously been included in a study focusing on tumor induced plasticity (22).

### Perioperative and intraoperative procedures

#### Imaging

MRI including tractography and neuronavigation sequences was done prior to surgery and postoperative MRI within 48 h after surgery according to our standard glioma imaging practice (23, 24). Shortly, a conventional MRI protocol consisting of 3D T2W, 3DT2-FLAIR (slice thickness, 1 mm), diffusion sequences, and pre- and post-contrast 3DT1w were acquired. Morphological MRI sequences were used to assess brain tumor location and heterogeneity, mass effect, contrast enhancement, and the presence of multiple brain lesions (23–25). T2 turbo spin echo or T2 FLAIR images in Vue picture archiving and communication system software (version 11.1.0, Carestream Health Inc., Rochester, NY) were used to segment the lesions both pre- and postoperatively with the aid of a semiautomatic method (Livewire algorithm) (26).

Morphologic and diffusion MRI of the brain was performed on a 3-T MRI scanner (Philips Achieva, Best, The Netherlands). DTI was performed using a single-shot spin echo sequence with echo-planar imaging, 60 contiguous slices, voxel size of 2 × 2 × 2 mm, echo time/repetition time of 77 ms/6,626 ms, a diffusion-weighting factor b = 1,000 s/mm^2^, and diffusion encoding along 48 directions. Motion and eddy current correction of acquired DTI data were automatically performed in BrainEx (NordicNeuroLab AS, Bergen, Norway). The parametric maps of fractional anisotropy, axial, and radial diffusivity were calculated and merged on T2-FLAIR volumetric sequences. Streamline tractography was performed with a fractional anisotropy threshold of 0.1, an angular threshold of 45°, and a minimum length of 20 mm. BrainEx was used for the placement and drawing of regions of interest (ROIs) and regions of avoidance (ROAs). The anatomical placement of the ROIs and ROAs was manually performed using the most validated DTI atlases as references (27–29). Using a two-ROI approach, inferior fronto-occipital fasciculus, superior longitudinal fasciculus [both horizontal indirect component (horizontal superior longitudinal fascicle) and vertical indirect component (vertical superior longitudinal fascicle)], arcuate fasciculus, cortico-spinal tract, frontal aslant tract, and optic radiation were reconstructed in each patient on the hemisphere of interest. MRI results were uploaded into a neuronavigation system (Brainlab, Munich, Germany). Intraoperatively, each of the positive functional points detected with subcortical PS or STS was acquired with the neuronavigation system, and the linear distance between eloquent points and reconstructed white matter pathways was assessed postoperatively. The measurement was recorded for each point on three axes (axial, coronal, and sagittal). The shortest anatomical distance to one or multiple white matter pathways was estimated in a blinded fashion for functional information related to the points.

#### Preoperative evaluation

Patients were assessed by a speech therapist and a neuropsychologist before surgery. The linguistic evaluation contained confrontation naming, language comprehension, phonological and semantic word fluency, tests of reading and writing, and phonological ability. Suitable intraoperative tasks were determined in a final preoperative team meeting. Patients treated with anti-epileptic drugs prior to surgery continued with the ordinary dose.

#### Anesthesia and surgical technique

The anesthetic technique was according to an asleep-awake-sedation/asleep protocol. Intravenous propofol, remifentanil, and dexmedetomidine were used for general anesthesia and sedation. The airways were secured with a laryngeal mask in the asleep state. A bolus of intravenous fosphenytoin (15 mg/kg) was given for intraoperative seizure prophylaxis. The corticectomy started after the cortical mapping and the resection of subcortical structures was continuous until functional limits were detected, leaving the pathological tissue *in situ*. This reduced the possibility to anatomical shift between the cortical and subcortical eloquent points. After registration of the eloquent points, the rest of the tumor was resected with ultrasonic dissector to reveal the medial or deep functional limit of the resection. In case of brain shift, an intraoperative navigated ultrasound (Flex focus 800, BK Medical, Denmark) probe was used to adjust the navigation accuracy as previously described (30–32).

#### Penfield stimulation and monitoring procedures

Cortical PS was performed in agreement with previous published recommendations for eloquent language mapping in the awake patient (9, 33). In brief, electrical stimulation (60 Hz, biphasic pulses with a 1-ms duration for 3 s) was performed using a bipolar probe with an interelectrode distance of 5 mm (Dr. Langer Medical GmbH, Waldkirch, Germany). The PS intensity (mA) for cortical and subcortical mapping was the same and set to a level that produced clinical effects from either ventral prefrontal or primary motor cortex stimulation. Of note, the PS intensity was never changed during subcortical mapping. Language-related tests were performed during electrical stimulation and for evaluating baseline language performance. Typically, a PC monitor in front of the patient displayed test-related images at a constant pace (typically 4.5–5 s per image). Electrical stimulation was controlled from a neuromonitoring device (Cadwell Industries, Washington, USA), which also recorded cortical electroencephalography (EEG), free-running electromyography (EMG), and MEP. Cortical EEG was used to detect seizure activity or after-discharges. Free-running EMG was used to detect stimulus-induced muscle contractions from PS or seizures. MEP recordings were used for subcortical motor mapping. Live recordings of neurophysiological signals and video/sound streams from the patient and the surgical microscope were saved for postoperative analysis.

#### Subcortical short-train stimulation and study protocol

Continuous (3-Hz) cathodal STS (five monophasic pulses, 4-ms interpulse interval, and 0.5-ms pulse duration) was delivered *via* the tip of a suction probe (Inomed, Medizintechnik GmbH, Emmendingen, Germany). A corkscrew scalp electrode (the STS anode) was placed in the sterile field toward the midline. STS was started when surgery reached subcortical structures. Subcortical STS was temporarily turned off in brain regions that were considered non-eloquent or during surgical procedures irrelevant for STS mapping (e.g., hemostasis). STS artifacts commonly obscured the EEG monitoring trace, and the stimulation was intermittently paused for a few seconds to detect possible after-discharges or to exclude non-convulsive seizure as an explanation for language errors or other behavioral alterations.

Tumor resection was interrupted if language testing revealed language disturbance while running STS. As a first step, STS was canceled to confirm that the deterioration was a stimulation effect. If so, the next step was to restart STS and more precisely locate the area where stimulation produced errors and to establish the threshold stimulus intensity for language interference. The stimulating current was gradually lowered from a maximum intensity of 15 mA and stepwise down to a minimum of 2.5 mA (range, 15–10–7.5–5.0–2.5 mA) as long as language performance was affected by the stimulation. For example, if language errors occurred at 7.5 mA but not 5 mA, then 7.5 mA was considered the stimulus threshold for language interference at that location. Finally, fixed intensity PS was performed at the same anatomical site for comparison. Alternatively, if there were no language deficits from continuous STS, then PS was carried out according to routine (e.g., near eloquent subcortical tracts according to brain anatomy, fiber tracking, or MR findings) or the study protocol (at regular time intervals). STS was then tried at the same anatomical site for comparison with PS.

#### Data analysis

Subcortical locations in which monopolar STS effects were systematically validated by bipolar PS were separated into three groups. Group 1 represented locations where STS did not produce language disturbance. Groups 2 and 3 represented subcortical locations where STS produced language interference at two different stimulation intensity (threshold) levels (≥7.5 and ≤5 mA, respectively). PS effects regarding language interference were classified as either positive or negative. PS was defined as positive if two out of three stimulation trials produced errors in concurrent language tests. STS was classified as positive if the patient presented language errors or a marked language performance deterioration from stimulation. The Kruskal–Wallis statistic test was used to analyze whether the proportion of PS interference (positive PS) differed between the groups.

## Results

### Clinical results

The clinical characteristics of the 14 patients are described in **Table 1**. In summary, six men and eight women were included. The mean age (SD) was 48.1 (16.7) (min of 18 to max of 71) years. All tumors were left-sided. Median interquartile range (IQR) tumor volume was 39.5 (18.1–58.6) cm^3^ and grade of resection 92.0% (77.0%–96.0%). Eleven patients had preoperative language impairment. One patient was left-handed (patient 13), and functional MRI investigation displayed bilateral language organization but dominant left-sided activation.

**Table 1:** Patient characteristics.

Patient	Gender	Age	Handedness	Location (left side)	Diagnosis (WHO grade)	Tumor volume (cm^3^)	EOR (%)	Preoperative language impairment
1	Female	56	Right	Frontal pole/anterior cingulum	O3	18.1	97	Yes
2	Female	55	Right	HIPP/PHG	A3	28.0	91	Yes
3	Male	44	Right	Temporo-insular, STG, MTG	O2	56.1	95	No
4	Female	71	Right	SMG, MTG	GBM 4	9.3	81	Yes
5	Female	27	Right	ITG, PHG	A2	66.2	93	Yes
6	Male	45	Right	Fronto-insular	A2	46.5	94	No
7	Male	69	Right	DLPFC	A3-4	27.5	90	Yes
8	Female	51	Right	Fronto-insular, CR, SMA	O2	57.0	20	Yes
9	Female	37	Right	Fronto-temporo-insular	O3	202	77	Yes
10	Male	24	Right	Parietal	A3	58.6	75	No
11	Male	56	Right	Frontal-SMA	O3	32.4	96	Yes
12	Female	52	Right	Fronto-temporo-insular	O2	15.9	99	Yes
13	Female	68	Left	Fronto-temporo-insular	A3	83.9	75	Yes
14	Male	18	Right	Temporo-occipital	G1	9.5	100	Yes

A2, astrocytoma WHO grade 2; A3, astrocytoma WHO grade 3; CR, corona radiata; DLPFC, dorsolateral prefrontal cortex; G1, ganglioglioma WHO grade 1; GBM, glioblastoma multiforme WHO grade 4; EOR, extent of resection; ITG, inferior temporal gyrus; HIPP, hippocampus; O2, oligodendroglioma WHO grade 2; O3, oligodendroglioma WHO grade 3; MTG, middle temporal gyrus; PHG, parahippocampal gyrus; SMA, supplementary motor area; SMG, superior marginal gyrus; STG, superior temporal gyrus.

### Stimulation results

#### Language interference from STS and comparison with PS

Stimulation data from 76 subcortical STS–PS comparison sites were analyzed (median, five sites per patient at different locations and from all patients). After offline review of the videos, 70 sites remained for analysis after discarding six sites due to highly unstable baseline patient performance. Continuous (3-Hz) STS was started when the surgery reached subcortical structures. The STS intensity for probing subcortical matter varied depending on the surgical situation, e.g., moving to a new subcortical area at higher trial intensities (10–15 mA) or proceeding further toward eloquent structures at lower intensities. If STS produced language errors, then the minimal (threshold) STS current intensity required to induce interference was established. PS was carried out subsequently for comparison. Language interference from PS (positive PS) was considered indicative of close proximity to language tracts.

In accordance with the study protocol and to rule out false-negative STS results, PS was tested in 28 subcortical locations where continuous STS (median, 10 mA; range, 7.5–10 mA) did not interfere with language (Group 1, **Table 2**). PS was negative in all locations indicating reliable “negative mapping”. In the remaining 42 locations, STS produced language interference (median, three locations per patient). As shown in **Table 2**, language interference from STS at ≥ 7.5 mA (Group 2; median threshold, 10 mA; range, 7.5–15 mA) was typically not accompanied with positive PS. In contrast, the proportion of positive PS was considerable larger (~60%, p < 0.01) in Group 3 where STS interfered with language at lower intensities (≤ 5 mA; median, 5 mA; range, 2.5–5 mA). **Table 2** also shows that low intensity STS (≤ 5 mA) in Group 3 also produced language errors in locations where PS was negative (which possibly represented “false-positive” findings). The median PS intensity for all 70 test locations was 4 mA (range, 4–6 mA).

**Table 2:** Distribution of PS and STS effects.

		Group 1	Group 2	Group 3
PS	Positive	0	1 (6%)	15 (62%) *
	Negative	28 (100 %)	17 (94%)	9 (38%)
	**Total**	**28**	**18**	**24**

Group 1 represents location where STS (median 10 mA) did not produce language errors. Groups 2 and 3 represent locations where STS produced language interference at ≥7. 5 mA and ≤5 mA, respectively. The effects from PS (positive or negative) at corresponding subcortical locations are presented in the table. Asterisk (*) indicates that language interference from PS was statistically (p < 0.01) more prevalent in Group 3.

#### Stimulation effects and distance to subcortical fiber tracts

**Table 3** shows that subcortical STS at ≤ 5 mA and PS produced similar types of language errors in most subcortical locations (group 3). **Table 3** also shows that STS language interference occurred when the monopolar suction probe was 0–5 mm from language tracts in 14 locations (60%), 6–10 mm in four locations (17%), and >10 mm in the remaining five locations (23%). Apparently, there were no consistent findings concerning language interference from low intensity STS (≤ 5 mA) and nearby (≤ 5 mm) language-related tracts based on DTI.

**Table 3:** Language interference and probe distance to language tracts.

Patient#	Test	Error (STS)	Error (PS)	Tract (mm)
3	Rep	Phonological	Phonological	>10 mm
	Rep	Phonological	Phonological	IFOF (6)
	Rep	Anomia	Anomia Phonological	IFOF (0)
	Rep	Anomia Phonological	Phonological	IFOF (3)
	Rep	Anomia	Anomia	n/a
6	Obj	Delay Semantic	–	IFOF (2)
7	Obj	Anomia	Anomia	hSLF (4), FAT (5)
	Obj	Delay	–	FAT (3), IFOF (4), hSLF (9)
8	Obj	Anomia Semantic	Delay Anomia	AF (6), IFOF (10)
	Obj	Anomia	–	IFOF (0)
	Obj	Anomia	–	IFOF (0)
	Obj	Latency	–	IFOF (0), AF (4)
	Obj	Latency	–	AF (3), IFOF (8)
	Obj	Anomia	Anomia Delay Semantic	IFOF (0)
9	Rep	Phonological	–	>10 mm
10	Obj	Verbal apraxia	–	>10 mm
	Obj	Latency Anarthria	Delay Anarthria	>10 mm
	Obj	Verbal apraxia	–	>10 mm
12	Obj	Verbal apraxia	Verbal apraxia	7 hSLF, 9 AF
	Obj	Dysarthria Slow speech	Dysarthria Anarthria	8 vSLF
	Obj	Dysarthria	Dysarthria Slow speech	0 AF, 0 hSLF, 0 IFOF
	Obj	Dysarthria Verbal apraxia	Dysarthria Verbal apraxia	0 AF, 0 hSLF, 3 IFOF
	Obj	Dysarthria	Dysarthria	0 hSLF, 2 AF, 6 IFOF
	Obj	Neologism Perseveration	Lipothymia	3 IFOF, 5 AF, 5 hSLF

Comparison between STS (≤ 5 mA) and PS effects at 24 subcortical locations. The DTI-estimated distance to language tracts is given in millimeters (mm). # indicates patient number given in **Table 1**. AF, arcuate fascicle; hSLF, horizontal superior longitudinal fascicle; IFOF, inferior fronto-occipital fascicle; vSLF, vertical superior longitudinal fascicle. Rep, word repetition; Obj, object naming; Phonological, phonological paraphasia; Semantic, semantic paraphasia.

#### Side effects from electrical stimulation

One patient had a short self-limited focal seizure, and five patients had EEG after-discharges from cortical PS. The after-discharges disappeared either spontaneously or after cortical irrigation with cold Ringer solution. There were, however, no seizures or EEG after-discharges from subcortical stimulation (PS or STS). In five patients (nos. 2–6), STS produced ipsilateral facial muscle contractions that limited the maximum current strength to typically 7.5 mA in those situations. Three patients had occasional pain or discomfort during subcortical STS that seemed to emanate from the “return current” anodal corkscrew electrode that was secured to the scalp. Additional infiltration of the surrounding scalp area with local anesthetics was sometimes required. Another side effect, apparent in the review of some intraoperative video recordings, was related to the manual handling of the suction probe. Sweeping probe movements in the resection cavity (for suction purposes and during ongoing stimulating) seemed to produce language errors that were difficult to separate from spontaneous deterioration in baseline performance. This phenomenon sometimes occurred in areas where previous (or later) stimulation trials—with the probe held in a steady position—produced language interference.

## Discussion

In a recent methodological review on awake language mapping, Morshed et al. (21) suggested that continuous stimulation from a suction probe (16) may be useful for subcortical language mapping as it allows for simultaneous stimulation and resection. To our knowledge, however, this is the first detailed report on the actual implementation of this technique in awake surgery. With the *a priori* assumption that language interference from PS indicates close proximity (2–5 mm) to language tracts (8, 33), we were able to demonstrate that language interference from subcortical STS at low intensities (≤5 mA) more likely represent close proximity to language tracts than if higher STS intensities (≥7.5 mA) are required to produce errors. These findings may seem rather expected considering current knowledge about the relationship between subcortical monopolar STS current intensity required to produce MEPs and the distance to corticospinal tracts (1 mA to ~1 mm) in the asleep patient (15, 34). In addition, in subcortical motor mapping with monopolar STS, it has been proposed that glioma resection may continue as long as the minimal intensity required to elicit motor responses is above 1–5 mA (35, 36). The relationship (1 mA to ~1 mm) and the proposed safety limit (2–5 mA) for subcortical motor mapping explain our rationale for separating the STS trial intensities into two groups (≤5 and ≥7.5 mA). Intensities between 5 and 7.5 mA were not examined for practical reasons (time constraint). On the basis of the STS–PS comparison, we infer that the minimal monopolar STS intensity required to produce language interference is related to the distance to subcortical language tracts, although we were not able to confirm this by DTI tractography. In the few previous studies (17, 18) exploring monopolar “high-frequency” stimulation (i.e., STS) in awake glioma surgery, the same current intensity applied for cortical mapping was also used for subcortical stimulation. On the basis of our results, and in agreement with the experience with monopolar STS subcortical motor mapping, this strategy may potentially produce “long-range” language interference at high stimulus intensities, which does not necessarily indicate proximity to language-related tracts. Finally, we found that continuous (3-Hz) cathodal subcortical STS delivered *via* a suction probe was easy to implement and well tolerated by the awake patient throughout surgery. The only apparent side effects were related to transient current spread to the facial nerve and discomfort from the subcortical STS “return current” scalp anodal electrode. No seizures were detected either clinically or in the electrocorticography from subcortical stimulation.

### Reliability

If monopolar subcortical STS gains popularity in awake surgery, then more information on the reliability (e.g., sensitivity and specificity) of detecting eloquent subcortical structures will hopefully accumulate. The only previous study (17) comparing STS with conventional PS demonstrated (in 50 patients) that the rate of errors (articulatory, anomia, and paraphasia) from electrical stimulation was not significantly different between the two methods. Our study (exploring continuous STS from a suction probe) lacks statistical power to draw any conclusions on whether STS is sufficiently reliable to replace PS. Nevertheless, in agreement with Riva et al. (17), we also found that STS and PS in general produced the same type of language errors (e.g., anomia or no response) in the same subcortical locations (**Table 3**). On the other hand, there were also a considerable number of locations in which STS language interference (at ≤ 5 mA) was not confirmed by PS. As the sensitivity for PS in detecting eloquent structures appears to be very high (33), this observation may indicate potential false-positive STS findings, i.e., language errors not related to the stimulated area under the probe. Despite our best efforts, misinterpretation of stimulation effects in some locations cannot be ruled out because of temporary deterioration in baseline performance from exhaustion/drowsiness, surgical manipulation, or ultrasound aspiration. Further, current spread/shunting through fluid toward more distant subcortical structures might be a particular problem with a monopolar stimulation technique, perhaps even at lower stimulation strengths (≤5 mA). At somewhat higher intensities (≥7.5 mA), this phenomenon was clearly manifested as remote ipsilateral facial nerve depolarization as already described above. Alternatively, it is possible that STS language interference in combination with negative PS at some locations (**Table 3**) was due to failed PS because of inappropriate orientation of the bipolar PS probe along the subcortical tracts or current shunting through fluid between the 5-mm spaced anode and cathode (i.e., short circuit) (33).

Continuous subcortical STS and unaffected patient language performance seemed to represent reliable “negative mapping” as judged by control stimulation with PS that were negative in all of the STS negative locations. Obviously, such results are expected in tumor areas remote from language-related fibers. However, our comparisons were also made as the tumor resection gradually approached vital/anatomical “borders” with different stimulation scenarios, as for example, negative mapping or STS interference at ≥7.5 mA (and negative PS) that allowed for further resection.

### Distance assessment to language-related tracts

We were not able to establish the type of “current strength (mA) – distance (mm) to eloquent tract” relationship that is available for subcortical motor mapping. There were inconsistent results when the relationship between STS effects at low intensities (≤ 5 mA) and DTI findings was analyzed (**Table 3**). DTI confirmed language-related tracts close (0–5 mm) to the stimulation spot in only 14 out of 23 positive STS sites (≤ 5 mA). We also found that some of those sites where subcortical stimulation interfered with language-related tasks did not harbor language-related tracts according to preoperative DTI. These inconsistencies may be due to the intrinsic limitation of DTI reconstructions (37–41). Our tractography results are based on the fiber assignment by continuous tracking (FACT) algorithm that is considered suboptimal in the reconstruction of white matter bundles close to kissing–crossing fiber regions compared with other techniques (39). In addition, large tumor volume, peritumoral edema, or previous radiotherapy usually affects the reconstruction of peritumoral white matter pathways, which may result in incomplete reconstruction (38, 42). Moreover, despite the possibility of correcting the brain-shift effect with ultrasound, we cannot exclude a possible imprecise acquisition of eloquent spots. Therefore, the pre- and postoperative comparison of tractographic results should be cautiously interpreted. Nevertheless, we point out that the DTI results provide complementary anatomical information and better surgical planning with possible predictions of anatomical–functional networks to test during our resection (37, 38).

### Side effects

Despite the frequent stimulation (theoretically ~11,000 bursts of electrical pulses per hour), there were no stimulus-induced seizures from subcortical STS in the awake patient. There are several possible explanations for this outcome. For example, subcortical stimulation, in contrast to cortical stimulation, is less likely to induce seizures, and the stimulation was commonly paused when the tumor resection approached cortical areas. Occasionally, the stimulating tip of the suction probe came in contact with cortical structures without producing seizures, possibly because STS seems to be a low-level ictogenic cortical stimulation technique, at least compared with PS (17, 43). Perhaps, more importantly, all patients were loaded with anticonvulsant fosphenytoin (15 mg phenytoin sodium equivalents (PE)/kg) at the beginning of the surgery (43).

The most obvious side effect (but a minor problem for the patient) was that, in several patients, the monopolar STS produced depolarization of the ipsilateral facial nerve with corresponding rhythmical (3-Hz) twitches of the facial muscles. This phenomenon, which typically occurred in inferior or basal temporal brain regions, was easy to clinically identify and was not encountered at lower (≤7.5 mA) stimulation strengths. The maximum initial intensity was consequently set to 10 mA in most patients to minimize the problem. Another side effect was that a few patients complained about repetitive (i.e., 3 Hz) throbbing or pain sensations in the scalp that were clearly related to the monopolar STS. It is important to realize that the anodal scalp electrode—required for subcortical cathodal STS—also acts as a stimulating electrode of cutaneous scalp tissue. Therefore, this electrode should be available for additional infiltration with local anesthetics. We also learned that continuous STS sometimes interfere with baseline language testing as the suction tip occasionally reaches previously established eloquent sites in the resection cavity. Moreover, current spread from irrigation may reach nearby subcortical language fibers outside the immediate resection area and may account for “false-positive” STS findings. Although using a suction device as an electrical stimulator seems ideal in this context, it is advisable to pause the continuous stimulation to allow for potential recovery before further subcortical mapping. In our current practice, we commonly switch from continuous to intermittent subcortical STS when baseline performance is unstable.

### Conclusion

Repetitive (3-Hz) STS delivered *via* a suction probe is feasible throughout awake subcortical glioma resection. The monopolar STS current (mA) intensity required for language interference seems to provide information about the distance to language-related tracts. We were not able to prove this directly with DTI tractography. Instead, we used PS as a surrogate marker (positive or negative) to confirm close proximity to language-related tracts. We demonstrated that language interference from STS at low intensities (≤ 5 mA)—to a large extent—produced language errors similar to PS in the same subcortical areas. In contrast, STS language interference at ≥7.5 mA or no effect from the stimulation was typically accompanied with negative PS, which seemed to indicate safe distance to language-related tracts. There may have been “false-positive” STS results in our study, which—together with other limitations (limited sample size and awake test battery)—suggest that subcortical bipolar PS should be available for validation. We point out that the prime role for continuous STS is to act as a “scanner”, where no language interference during stimulation, or interference only at “higher” intensities, allows for safe resection toward functional boundaries. This technique could be a time-saving strategy that is particularly important in awake surgery where neuroimaging may be inaccurate and gradual patient exhaustion influences baseline language performance and test cooperability. Continuous scanning with STS may also enable early detection of language-related tracts and where PS is used for confirmation.

## Data availability statement

The datasets presented in this article are not readily available because Data in the form of numbers and text are available but not video recordings of patients. Requests to access the datasets should be directed to Hans Axelson, hans.axelson@akademiska.se.

## Ethics statement

The studies involving human participants were reviewed and approved by The regional Ethics committee of Uppsala (registrator@etikprovning.se). The patients/participants provided their written informed consent to participate in this study.

## Author contributions

All authors contributed to conception and design of the study. HA wrote the first draft of the manuscript. HA and MJ reviewed intraoperative video recordings. HA, FL, MJ and MZ wrote sections of the manuscript. All authors contributed to manuscript revision, read, and approved the submitted version

## Funding

This research was partly funded by grants from the non-profit Margaretahemmet society.

## Acknowledgments

The authors thank engineer Mats Jutterström for assisting in the development of the custom-made language testing equipment, technicians Carolina Brink and Malin Garret for meticulous written records of stimulation effects, and neuropsychologist Åsa Alberius Munkhammar for assisting in the intraoperative testing.

## Conflict of interest

The authors declare that the research was conducted in the absence of any commercial or financial relationships that could be construed as a potential conflict of interest.

The handling editor GT declared a past co-authorship with the authors FL, MJ, MZ, MR.

## Publisher’s note

All claims expressed in this article are solely those of the authors and do not necessarily represent those of their affiliated organizations, or those of the publisher, the editors and the reviewers. Any product that may be evaluated in this article, or claim that may be made by its manufacturer, is not guaranteed or endorsed by the publisher.

## References

[1] Capelle L Fontaine D Mandonnet E Taillandier L Golmard JL Bauchet L. Spontaneous and therapeutic prognostic factors in adult hemispheric world health organization grade II gliomas: a series of 1097 cases: clinical article. J Neurosurg (2013) 118(6):1157–68. doi: 10.3171/2013.1.JNS121 23495881

[2] McGirt MJ Chaichana KL Attenello FJ Weingart JD Than K Burger PC. Extent of surgical resection is independently associated with survival in patients with hemispheric infiltrating low-grade gliomas. Neurosurgery. (2008) 63(4):700–7; author reply 7-8. doi: 10.1227/01.NEU.0000325729.41085.73 18981880

[3] Sanai N Berger MS. Glioma extent of resection and its impact on patient outcome. Neurosurgery (2008) 62(4):753–64; discussion 264-6. doi: 10.1227/01.neu.0000318159.21731.cf 18496181

[4] Smith JS Chang EF Lamborn KR Chang SM Prados MD Cha S. Role of extent of resection in the long-term outcome of low-grade hemispheric gliomas. J Clin Oncol (2008) 26(8):1338–45. doi: 10.1200/JCO.2007.13.9337 18323558

[5] Duffau H Lopes M Arthuis F Bitar A Sichez JP Van Effenterre R. Contribution of intraoperative electrical stimulations in surgery of low grade gliomas: a comparative study between two series without (1985-96) and with (1996-2003) functional mapping in the same institution. J Neurol Neurosurg Psychiatry (2005) 76(6):845–51. doi: 10.1136/jnnp.2004.048520 PMC1739650 15897509

[6] Soffietti R Baumert BG Bello L von Deimling A Duffau H Frenay M. Guidelines on management of low-grade gliomas: report of an EFNS-EANO task force. Eur J Neurol (2010) 17(9):1124–33. doi: 10.1111/j.1468-1331.2010.03151.x 20718851

[7] Penfield W Baldrey E. Somatic motor and sensory representationin the cerebral cortex of man as studied by electrical stimulation. Brain. (1937) 60:389–443. doi: 10.1093/brain/60.4.389

[8] Duffau H. Stimulation mapping of white matter tracts to study brain functional connectivity. Nat Rev Neurol (2015) 11(5):255–65. doi: 10.1038/nrneurol.2015.51 25848923

[9] Gogos AJ Young JS Morshed RA Hervey-Jumper SL Berger MS. Awake glioma surgery: technical evolution and nuances. J Neurooncol (2020) 147:515–24. doi: 10.1007/s11060-020-03482-z 32270374

[10] Keles GE Lundin DA Lamborn KR Chang EF Ojemann G Berger MS. Intraoperative subcortical stimulation mapping for hemispherical perirolandic gliomas located within or adjacent to the descending motor pathways: evaluation of morbidity and assessment of functional outcome in 294 patients. J Neurosurg (2004) 100(3):369–75. doi: 10.3171/jns.2004.100.3.0369 15035270

[11] Kombos T Suess O Kern BC Funk T Hoell T Kopetsch O. Comparison between monopolar and bipolar electrical stimulation of the motor cortex. Acta Neurochir (Wien) (1999) 141(12):1295–301. doi: 10.1007/s007010050433 10672300

[12] Szelenyi A Senft C Jardan M Forster MT Franz K Seifert V. Intra-operative subcortical electrical stimulation: a comparison of two methods. Clin neurophysiol: Off J Int Fed Clin Neurophysiol (2011) 122(7):1470–5. doi: 10.1016/j.clinph.2010.12.055 21330203

[13] Sala F. Penfield’s stimulation for direct cortical motor mapping: An outdated technique? Clin Neurophysiol (2018) 129(12):2635–7. doi: 10.1016/j.clinph.2018.09.021 30424944

[14] Nossek E Korn A Shahar T Kanner AA Yaffe H Marcovici D. Intraoperative mapping and monitoring of the corticospinal tracts with neurophysiological assessment and 3-dimensional ultrasonography-based navigation. Clin Article J Neurosurg (2011) 114(3):738–46. doi: 10.3171/2010.8.JNS10639 20799862

[15] Ohue S Kohno S Inoue A Yamashita D Harada H Kumon Y. Accuracy of diffusion tensor magnetic resonance imaging-based tractography for surgery of gliomas near the pyramidal tract: a significant correlation between subcortical electrical stimulation and postoperative tractography. Neurosurgery (2012) 70(2):283–93; discussion 94. doi: 10.1227/NEU.0b013e31823020e6 21811189

[16] Raabe A Beck J Schucht P Seidel K. Continuous dynamic mapping of the corticospinal tract during surgery of motor eloquent brain tumors: evaluation of a new method. J Neurosurg (2014) 120(5):1015–24. doi: 10.3171/2014.1.JNS13909 24628613

[17] Riva M Fava E Gallucci M Comi A Casarotti A Alfiero T. Monopolar high-frequency language mapping: can it help in the surgical management of gliomas? a comparative clinical study. J Neurosurg (2016) 124(5):1479–89. doi: 10.3171/2015.4.JNS14333 26406788

[18] Verst SM de Aguiar PHP Joaquim MAS Vieira VG Sucena ABC Maldaun MVC. Monopolar 250-500Hz language mapping: Results of 41 patients. Clin Neurophysiol Pract (2019) 4:1–8. doi: 10.1016/j.cnp.2018.11.002 30619979 PMC6312792

[19] Axelson HW Hesselager G Flink R. Successful localization of the broca area with short-train pulses instead of ‘Penfield’ stimulation. Seizure (2009) 18(5):374–5. doi: 10.1016/j.seizure.2009.01.005 19201212

[20] Rossi M Sani S Nibali MC Fornia L Bello L Byrne RW. Mapping in low-grade glioma surgery: Low- and high-frequency stimulation. Neurosurg Clin N Am (2019) 30(1):55–63. doi: 10.1016/j.nec.2018.08.003 30470405

[21] Morshed RA Young JS Lee AT Berger MS Hervey-Jumper SL. Clinical pearls and methods for intraoperative awake language mapping. Neurosurgery (2021) 89(2):143–53. doi: 10.1093/neuros/nyaa440 PMC8279835 33289505

[22] Latini F Axelson H Fahlström M Jemstedt M Alberius Munkhammar Å Zetterling M. Role of preoperative assessment in predicting tumor-induced plasticity in patients with diffuse gliomas. J Clin Med (2021) 10(5). doi: 10.3390/jcm10051108 PMC7961995 33799925

[23] Freyschlag CF Krieg SM Kerschbaumer J Pinggera D Forster MT Cordier D. Imaging practice in low-grade gliomas among European specialized centers and proposal for a minimum core of imaging. J Neurooncol (2018) 139(3):699–711. doi: 10.1007/s11060-018-2916-3 29992433 PMC6132968

[24] Thust SC Heiland S Falini A Jager HR Waldman AD Sundgren PC. Glioma imaging in Europe: A survey of 220 centres and recommendations for best clinical practice. Eur Radiol (2018) 28(8):3306–17. doi: 10.1007/s00330-018-5314-5 PMC6028837 29536240

[25] Dodo T Okada T Yamamoto A Kanagaki M Fushimi Y Okada T. T1-weighted MR imaging of glioma at 3T: a comparative study of 3D MPRAGE vs. conventional 2D spin-echo imaging. Clin Imaging (2016) 40(6):1257–61. doi: 10.1016/j.clinimag.2016.08.016 27639863

[26] Latini F Larsson EM Ryttlefors M. Rapid and accurate MRI segmentation of peritumoral brain edema in meningiomas. Clin Neuroradiol (2017) 27(2):145–52. doi: 10.1007/s00062-015-0481-0 26603998

[27] Catani M Thiebaut de Schotten M. A diffusion tensor imaging tractography atlas for virtual *in vivo* dissections. Cortex. (2008) 44(8):1105–32. doi: 10.1016/j.cortex.2008.05.004 18619589

[28] Mori S Kaufmann WE Davatzikos C Stieltjes B Amodei L Fredericksen K. Imaging cortical association tracts in the human brain using diffusion-tensor-based axonal tracking. Magn Reson Med (2002) 47(2):215–23. doi: 10.1002/mrm.10074 11810663

[29] Wakana S Caprihan A Panzenboeck MM Fallon JH Perry M Gollub RL. Reproducibility of quantitative tractography methods applied to cerebral white matter. Neuroimage. (2007) 36(3):630–44. doi: 10.1016/j.neuroimage.2007.02.049 PMC2350213 17481925

[30] Gerard IJ Kersten-Oertel M Petrecca K Sirhan D Hall JA Collins DL. Brain shift in neuronavigation of brain tumors: A review. Med Image Anal (2017) 35:403–20. doi: 10.1016/j.media.2016.08.007 27585837

[31] Ohue S Kumon Y Nagato S Kohno S Harada H Nakagawa K. Evaluation of intraoperative brain shift using an ultrasound-linked navigation system for brain tumor surgery. Neurologia Medico-Chirurgica (2010) 50(4):291–300. doi: 10.2176/nmc.50.291 20448420

[32] Rasmussen IA Jr. Lindseth F Rygh OM Berntsen EM Selbekk T Xu J. Functional neuronavigation combined with intra-operative 3D ultrasound: initial experiences during surgical resections close to eloquent brain areas and future directions in automatic brain shift compensation of preoperative data. Acta Neurochir (Wien) (2007) 149(4):365–78. doi: 10.1007/s00701-006-1110-0 17308976

[33] Pallud J Mandonnet E Corns R Dezamis E Parraga E Zanello M. Technical principles of direct bipolar electrostimulation for cortical and subcortical mapping in awake craniotomy. Neurochirurgie. (2017) 63(3):158–63. doi: 10.1016/j.neuchi.2016.12.004 28506482

[34] Kamada K Todo T Ota T Ino K Masutani Y Aoki S. The motor-evoked potential threshold evaluated by tractography and electrical stimulation. J Neurosurg (2009) 111(4):785–95. doi: 10.3171/2008.9.JNS08414 19199462

[35] Schucht P Seidel K Jilch A Beck J Raabe A. A review of monopolar motor mapping and a comprehensive guide to continuous dynamic motor mapping for resection of motor eloquent brain tumors. Neurochirurgie. (2017) 63(3):175–80. doi: 10.1016/j.neuchi.2017.01.007 28506487

[36] Asimakidou E Abut PA Raabe A Seidel K. Motor evoked potential warning criteria in supratentorial surgery: A scoping review. Cancers (Basel) (2021) 13(11). doi: 10.3390/cancers13112803 PMC8200078 34199853

[37] Duffau H. Diffusion tensor imaging is a research and educational tool, but not yet a clinical tool. World Neurosurg (2014) 82(1-2):e43–5. doi: 10.1016/j.wneu.2013.08.054 24017954

[38] Yu CS Li KC Xuan Y Ji XM Qin W. Diffusion tensor tractography in patients with cerebral tumors: a helpful technique for neurosurgical planning and postoperative assessment. Eur J Radiol (2005) 56(2):197–204. doi: 10.1016/j.ejrad.2005.04.010 15916876

[39] Farquharson S Tournier JD Calamante F Fabinyi G Schneider-Kolsky M Jackson GD. White matter fiber tractography: why we need to move beyond DTI. J Neurosurg (2013) 118(6):1367–77. doi: 10.3171/2013.2.JNS121294 23540269

[40] Maier-Hein KH Neher PF Houde JC Cote MA Garyfallidis E Zhong J. The challenge of mapping the human connectome based on diffusion tractography. Nat Commun (2017) 8(1):1349. doi: 10.1038/s41467-017-01285-x 29116093 PMC5677006

[41] Pujol S Wells W Pierpaoli C Brun C Gee J Cheng G. The DTI challenge: Toward standardized evaluation of diffusion tensor imaging tractography for neurosurgery. J Neuroimaging (2015) 25(6):875–82. doi: 10.1111/jon.12283 PMC4641305 26259925

[42] Zhang H Wang Y Lu T Qiu B Tang Y Ou S. Differences between generalized q-sampling imaging and diffusion tensor imaging in the preoperative visualization of the nerve fiber tracts within peritumoral edema in brain. Neurosurgery. (2013) 73(6):1044–53; discussion 53. doi: 10.1227/NEU.0000000000000146 24056318

[43] Dineen J Maus DC Muzyka I See RB Cahill DP Carter BS. Factors that modify the risk of intraoperative seizures triggered by electrical stimulation during supratentorial functional mapping. Clin Neurophysiol (2019) 130(6):1058–65. doi: 10.1016/j.clinph.2019.03.006 30930194

